# Titanium Dioxide Nanomaterials: Progress in Synthesis and Application in Drug Delivery

**DOI:** 10.3390/pharmaceutics16091214

**Published:** 2024-09-16

**Authors:** Fanjiao Zuo, Yameng Zhu, Tiantian Wu, Caixia Li, Yang Liu, Xiwei Wu, Jinyue Ma, Kaili Zhang, Huizi Ouyang, Xilong Qiu, Jun He

**Affiliations:** 1 State Key Laboratory of Component-Based Chinese Medicine, Tianjin University of Traditional Chinese Medicine, Tianjin 301617, China; zuofanjiao@163.com (F.Z.); yameng354@163.com (Y.Z.); wutiant@139.com (T.W.); lcxtcm@126.com (C.L.); yeeliuyang@163.com (Y.L.); wuxiwei2022@163.com (X.W.); 13163106179@163.com (J.M.); zkl101033@163.com (K.Z.); huihui851025@163.com (H.O.); 2 School of Traditional Chinese Medicine, Tianjin University of Traditional Chinese Medicine, Tianjin 301617, China

**Keywords:** titanium dioxide, synthesis methods, drug delivery systems

## Abstract

**Background:** Recent developments in nanotechnology have provided efficient and promising methods for the treatment of diseases to achieve better therapeutic results and lower side effects. Titanium dioxide (TiO_2_) nanomaterials are emerging inorganic nanomaterials with excellent properties such as low toxicity and easy functionalization. TiO_2_ with special nanostructures can be used as delivery vehicles for drugs, genes and antigens for various therapeutic options. The exploration of TiO_2_-based drug delivery systems shows great promise for translating nanotechnology into clinical applications; **Methods:** Comprehensive data on titanium dioxide were collected from reputable online databases including PubMed, GreenMedical, Web of Science, Google Scholar, China National Knowledge Infrastructure Database, and National Intellectual Property Administration; **Results:** In this review, we discuss the synthesis pathways and functionalization strategies of TiO_2_. Recent advances of TiO_2_ as a drug delivery system, including sustained and controlled drug release delivery systems were introduced. Rigorous long-term systematic toxicity assessment is an extremely critical step in application to the clinic, and toxicity is still a problem that needs to be closely monitored; **Conclusions:** Despite the great progress made in TiO_2_-based smart systems, there is still a great potential for development. Future research may focus on developing dual-reaction delivery systems and single-reaction delivery systems like redox and enzyme reactions. Undertaking thorough in vivo investigations is necessary prior to initiating human clinical trials. The high versatility of these smart drug delivery systems will drive the development of novel nanomedicines for personalized treatment and diagnosis of many diseases with poor prognosis.

## 1. Introduction

The drug delivery system (DDS) is an important area of biomedicine. Over the past decades, nanotechnology has played an increasingly important role in drug delivery [1,2]. Conventional DDSs (tablets, capsules, ointments, etc.) suffer from poor bioavailability, fluctuating plasma drug concentrations, and make it more difficult to control drug release [3,4]. Nanomaterials have excellent properties to meet the needs of smart DDSs, which are capable of aggregating at targeted sites such as tumor tissues, reducing systemic exposure and drug clearance through enhanced permeability and retention effects, and minimizing side effects [5,6,7].

Inorganic nanoparticles are one of the mainstream directions in nano-drug delivery research [8,9,10]. Inorganic nanomaterials, such as silica [11,12], titanium dioxide [13,14], manganese oxide [15], graphene [16], etc., are ideal for drug delivery due to their ease of preparation, high drug loading rate, excellent physicochemical properties, biocompatibility, and flexibility of surface modification (Table 1). In addition, inorganic nanomaterials have a wide range of applications in biochemical sensing [17], bioimaging [18], chemical catalysis [19], and other fields. In recent years, TiO_2_ nanomaterials and its composite materials have shown great potential in realizing targeted drug delivery, slow drug release, and improving the solubility and bioavailability of intolerable or insoluble drugs, and have also shown good application prospects in enhancing the therapeutic effects of diseases [20,21,22].

TiO_2_ nanomaterials with different structures exhibit varying characteristics [23,24,25]. To achieve high effectiveness in drug delivery applications, TiO_2_ with a wide surface area and porosity is typically required [26]. Various preparation methods are constantly being tried to obtain TiO_2_ with different mesoporous structures, pore wall parameters, morphologies and compositions. This review will focus on the recent preparation and functionalization developments of TiO_2_ and the last advances on their application in drug delivery technologies.

## 2. Synthesis of TiO_2_ Nanomaterials

Over the past few years, researchers have worked to improve the synthesis of TiO_2_ nanomaterials due to the demand for certain features and the shortcomings of current approaches. Among the several synthetic routes, the commonly used methods include the sol-gel method, hydrothermal synthesis, template method, gas-state method, and so on. There are significant differences in the size, shape, morphology, composition, mesoporous structure, specific surface area, and crystal structure of TiO_2_ prepared by different methods, which play a crucial role in the biomedical applications of nanoparticles. In this section, synthetic methods for TiO_2_ nanomaterials are summarized and compared (Table 2).

### 2.1. Sol-Gel Method

In the 1960s, the sol-gel method was used to prepare materials with various shapes and porous structures. The sol-gel procedure included the process of hydrolysis and polycondensation, during which process M-OH-M or M-O-M bridges were established between the atoms M of the precursor molecules, resulting in oxides or hydroxides. Specifically, for TiO_2_, titanium alkoxides (such as titanium (IV) isopropoxide, titanium butoxide), alcohol, and acid/water were introduced into the reaction system. After stirring for several hours, densely cross-linked three-dimensional structures were built and terminated as TiO_2_ gel. The reactions’ mechanism was as follows [27,28]:

The hydrolysis reactions:

(1) $$\mathrm{Ti}{\left(\mathrm{OR}\right)}_{n}+H_{2}O\to \mathrm{Ti}\left(\mathrm{OH}\right){\left(\mathrm{OR}\right)}_{n-1}+\mathrm{ROH}\;$$

(2) $$\mathrm{Ti}\left(\mathrm{OH}\right){\left(\mathrm{OR}\right)}_{n-1}+H_{2}O\to \mathrm{Ti}{\left(\mathrm{OH}\right)}_{2}{\left(\mathrm{OR}\right)}_{n-2}+\mathrm{ROH}.$$

The reactions continuously go on: ⋯ $\to \mathrm{Ti}{\left(\mathrm{OH}\right)}_{n}$.

The polycondensation reactions:

(3) $$\text{-}\mathrm{Ti}\text{-}\mathrm{OH}\;+\;\mathrm{HO}\text{-}\mathrm{Ti}\text{-}\;\to \;\text{-}\mathrm{Ti}\text{-}O\text{-}\mathrm{Ti}\text{-}\;+\;H_{2}O$$

(4) -Ti-OR+HO-Ti- → -Ti-O-Ti-+ROH. 

**Figure 1:**
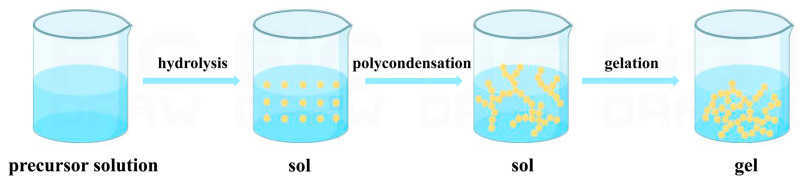
Schematic diagram of steps involved in sol-gel process.

After the sol-gel process, aging, drying and annealing were needed to create the final TiO_2_ nanoparticles [29]. Figure 1 shows a schematic diagram of the steps involved in the sol-gel process.

Sol-gel synthesis is one of the simplest, fastest, and economically less expensive methods, and has advantages like low processing temperature, homogeneity of the produced material, and formation of the complex structures or composite materials [30,31]. Gwanhee Park et al. added water in the form of steam in the reaction system, and the small droplets were dispersed to collide with the titanium source, which triggered hydrolysis reaction. The additional collision between the droplets was suppressed by excess anhydrous ethanol, which resulted in the formation of smaller nanoparticles than the ones in the conventional sol-gel method [32]. Jaroenworaluck et al. prepared anatase TiO_2_ nanoparticles up to 10 nm in size by mixing tetraisopropylorthotitanate, methanol, and ethanol in different molar ratios [33]. The excellent properties of the prepared nanoparticles were crucial in addition to their good morphology. Among the different crystalline phases of TiO_2_ nanoparticles, anatase is the key phase for photocatalysis [24,34]. Calabrese et al. developed reaction parameters based on the sol-gel method to promote local alignment of anatase particles, which created TiO_2_ with a tunable crystalline phase and excellent photocatalytic activity [35].

Apart from pure TiO_2_ nanoparticles, the researchers also synthesized TiO_2_ doped with different compositions using the sol-gel method, which greatly improved their properties for particular applications, such as photocatalysis, antimicrobial, and photovoltaic power generation [36,37,38]. Yadav et al. prepared Cu-, Fe-, Ni-, Cr-, and Co-doped TiO_2_ and undoped TiO_2_ nanostructures and demonstrated photocatalytic dye degradation and antibacterial application. Similarly, Shahina Kader et al. synthesized TiO_2_, MoO_3_/TiO_2_, Ag/TiO_2_ and MoO_3_/Ag/TiO_2_ photocatalysts and applied them in an immobilized form on the borosilicate glass surface to construct a reactor to evaluate the performance of degrading MO dyes under UV treatment [39].

### 2.2. Hydrothermal Method

The hydrothermal method referred to preparing TiO_2_ by utilizing the highly identical and anisotropic growth mode of crystals, dissolving titanium salts, titanium containing organic compounds or titanium oxide compounds in water, and reacting with additives such as alkali and dispersant in a closed system at a high temperature and high pressure. Hydrothermal synthesis involved three key steps, crystal growth, crystal transformation, and phase equilibrium, culminating in fine-to-ultrafine crystals [40]. By regulating the ratio of different titanium sources and additives, reaction temperature and reaction time, the nucleation rate and particle size distribution could be controlled, thus obtaining TiO_2_ nanomaterials with different morphologies.

The hydrothermal method had been used to prepare TiO_2_ by many research groups after the first introduction by Kasuga et al., which could produce TiO_2_ with homogeneity, high purity, crystal symmetry, metastable compounds with unique properties, and narrow particle size distributions [41]. In a typical synthesis, Thapa R et al. carried out a hydrothermal reaction with titanium isopropoxide, acetylacetone, and urea at 150 °C for 18 h, ultimately yielding TiO_2_ nanomaterials of anatase type, which proved to be an efficient catalyst [42]. Liu et al. prepared TiO_2_ hollow nanospheres by dissolving Ti (SO_4_)_2_ and NH_4_F in deionized water and conducting a hydrothermal reaction at 160 °C for 6 h. The synthesized TiO_2_ hollow nanospheres have high dispersibility and large specific surface area. Subsequently, porous hollow TiO_2_ nanospheres could be prepared by adding corrosive additives in the hydrothermal process, and their specific surface area was 168 m^2^·g^−1^ with an average pore size of 12 nm [43].

To fulfill more application needs and improve individual flaws, hydrothermal was used to continually create TiO_2_ with a new morphology and varied component doping [44,45]. Sun et al. produced eggshell-like TiO_2_ hollow sphere nanoparticles using a one-step template-free method, and investigated their electrorheological properties at various electric field strengths [46]. The Ti^3+^ self-doped TiO_2_ nanoparticles with a fusiform-like morphology were prepared via a facile mixed solvothermal method in a mixture of water and triethylamine [47]. Rani et al. investigated the effect of anionic and nonionic surfactants on the synthesis of core–shell Fe_3_O_4_@TiO_2_ nanocomposites by a hydrothermal process [48].

In recent years, the microwave-assisted hydrothermal method has been generally accepted by researchers. It is an economical, fast, green and effective synthetic strategy to prepare efficient nanomaterials with controllable particle size. Majid et al. studied Fe-doped TiO_2_ nanomaterials by microwave hydrothermal synthesis, which improved the band gap limitation of TiO_2_ in applications [49]. In addition, the microwave hydrothermal method was also characterized by a fast-heating rate, sensitive reaction, uniform heating, etc. [50]. The crystal size, morphology and agglomeration could be controlled by adjusting the ratio of precursor materials, pH of the reaction system, reaction time and temperature. Sahu, K et al. prepared Cu-doped TiO_2_ nanomaterials and investigated the optimum Cu-doping ratio. The optics of samples prepared after optimum Cu doping and electrical properties were substantially improved [51].

### 2.3. Template Method

The template method is often used to produce TiO_2_ with mesoporous or hollow structures, as the morphology of the pores can be well controlled. Based on the properties of the template employed in the reaction, the template method can be generally divided into two categories: soft template method and hard template method [52].

#### 2.3.1. Hard Template Method

The hard template method uses pre-synthesized organic or inorganic templates with specific shapes (morphology, surface curvature), which serve as molds for the replication of nanoporous/mesoporous TiO_2_ and do not involve any significant chemical interactions with the titanium precursors. The morphology of the resulting materials is pre-determined by the templates, which have well-defined nanostructures [53]. Silicon dioxide (SiO_2_) is widely utilized as a template; however, this has resulted in mesoporous structures that are similar to each other and unable to satisfy the increasingly demanding applications of functional replica materials. Evidently, the creation of a new template is the key to resolving this problem. Based on the successfully prepared hierarchical mesoporous SBA-15 silica microspheres with 2D and 3D porous structures [54], Wang et al. prepared hierarchical mesoporous TiO_2_ microspheres (HMM-TiO_2_-MSs) by the nanocasting method and optimized the process. The obtained HMM-TiO_2_-MSs reached a specific surface area and pore volume of 194 m^2^ g^−1^ and 0.68 cm^3^ g^−1^, respectively, which were more than twice those of the conventional SBA-15 templates [55]. In addition, Li et al. prepared photocatalysts with TiO_2_ and g-C_3_N_4_ multilayered inline-connected structures using natural montmorillonite as a hard template by two intercalation methods for the first time [56]. Simultaneously, using resorcinol/formaldehyde (RF) polymer resin as a hard template, Wang et al. synthesized hollow TiO_2_ spheres through the hydrolysis and carbonation of multifunctional kinetically controlled coatings [57]. The use of RF as a template reduced damage to the TiO_2_ structure during etching and required a few organic reagents, which was favorable in the context of green science. Furthermore, size-controllable hollow mesoporous TiO_2_ spheres with greater photocatalytic activity than TiO_2_ nanoparticles were created using carbon spheres as hard templates [58].

#### 2.3.2. Soft Template Method

During the soft-templating process, the precursors and surfactant templates can work together to assemble an ordered mesoporous structure, which is driven by the spontaneous trend of interface energy reduction [59]. In such cases, the obtained pore structures are determined by templating agents’ molecular properties as well as the synthetic conditions, such as reactant ratios, concentration, solvents, and temperature. The soft template method can be synthesized in both aqueous and non-aqueous solutions. Since titanium precursors are highly reactive and sensitive to moisture, hydrolysis is usually too fast for the aqueous route to be controlled, making polymerization very difficult. In contrast, the non-aqueous solution synthesis method, which is often referred to as evaporation-induced self-assembly (EISA), can effectively slow down the hydrolysis and condensation of titanium precursors. Organic molecular templates, also known as structural directing agents, are important for mesoporous structures due to their composition and characteristics. To date, cationic surfactants (e.g., quaternary ammonium salts), anionic surfactants (e.g., carboxylates, sulfates, and phosphates), and non-ionic surfactants (e.g., Pluronic P123 and F127) are the most often utilized templates [60]. Hung et al. prepared ordered hexagonal mesoporous TiO_2_ by the EISA method using Pluronic P123 and tetrabutyl orthotitanate (Ti (OBun)_4_, TBOT) as the templating agent and the titanium source, respectively. They elucidated the effects of surfactant concentrations on the pore arrangement, pore size, specific surface area and structure of mesoporous TiO_2_ by the EISA method [61]. The variation in block copolymer templates can lead to changes in the crystal structure of synthesized nanostructures, resulting in changes in activity [62].

Although the soft template method provides excellent control over mesoscopic structures, it lacks control over atomic-scale structures. Due to the usual decomposition of surfactants below 300 °C, mesoporous metal oxides (MMOs) are often destroyed during the crystallization process. As a result, MMOs is amorphous or semi-crystalline and has low thermal stability [63]. Recently, Xiong et al. established a universal polymer-oriented evaporation-induced self-assembly strategy for synthesizing MMOs with high crystallinity. In acidic hydration environments, metal chlorides are used as precursors for metal oxides, while the cationic polymers are used as porous precursors. This method solved the drawback of the classic sol-gel-based self-assembly route using amphiphilic block copolymers as templates, which was confined to amorphous or semi-crystalline MMOs with low thermal stability [64].

### 2.4. Gas-State Method

The gaseous process turns basic materials into a gaseous state via sublimation, evaporation, and degradation before growing crystals under supersaturated steam through condensation and crystallization. It is commonly used to deposit TiO_2_ nanoparticles on various substrates or create nanofilms [65,66,67]. It is mainly divided into physical vapor deposition (PVD) and chemical vapor deposition (CVD). PVD is a method of heating raw materials by physical means, such as evaporation, ionization or sputtering, and further condensing them to form a solid material, usually without involving chemical reactions [68,69,70]. The TiO_2_ nanomaterials prepared by this method have the advantages of high purity, uniform distribution, small particle size and good dispersion, and it is easy to control the thickness of the film. However, the disadvantage is that the prepared materials need to be deposited under vacuum conditions, the equipment is more expensive, and the operating costs are high.

Unlike PVD, CVD forms a solid layer with conformal and fine coverage on a substrate by a chemical reaction of gases. Othman S H et al. prepared TiO_2_ by CVD in the deposition temperature range of 300–700 °C. The crystalline structure of TiO_2_ could be controlled by adjusting the deposition temperature, and products with high crystallinity, good homogeneity and high photocatalytic efficiency were obtained [71]. Many attempts have been made to directly prepare or deposit TiO_2_ on different substrates by the CVD method [72,73,74], and it has been confirmed that the problem of aggregation, large bandgap and rapid recombination of photo-generated electron–hole pairs can be well solved [75,76,77,78].

### 2.5. Solid-State Method

Solid-state reactions are generally considered to be those in which the solid participates directly in the chemistry and undergoes a chemical change, or at least, a process occurring inside or outside the solid that has a controlling effect. TiO_2_ is typically obtained by thoroughly mixing, grinding and calcining the formulated titanium or titanium oxide. TiO_2_/γ-Fe_2_O_3_ nanocomposites were synthesized as nano-adsorbent through a ball milling process by Mercyrani et al. Variations in grinding time and ball-to-powder ratio allowed for the control of the properties of the composites. Characterization such as XRD showed that spherical anatase TiO_2_ and cubic γ-Fe_2_O_3_ were predominantly formed under the established method [79]. Cao et al. reported a facile solid-state method to synthesize anatase TiO_2_ single crystals with 35% exposure of (001) facets and high purity. The shape of the obtained single crystals of TiO_2_ is characteristic of truncated bipyramids, and their sizes varied from 2–3 μm to 50–200 nm. They believed that with the application of this synthetic technique, TiO_2_ single-crystal materials may now be prepared on a massive scale [80]. In terms of the solid-state method, it is easy to operate and yields a more considerable amount of the desired product with a short processing time at ambient conditions; however, there are certain disadvantages, including easy introduction of impurities, incomplete morphology and inhomogeneous particle size. Hence, it is more suitable for large-scale industrial production [81,82].

### 2.6. Green Synthesis Method

**Figure 2:**
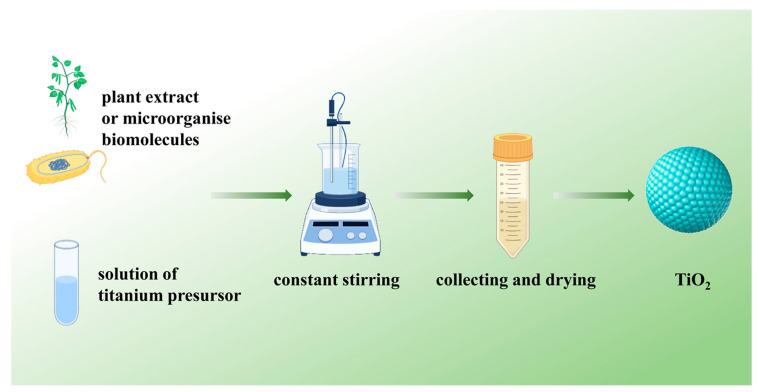
Schematic diagram of the preparation process of nanoparticles via the green synthesis method.

Although the sol-gel, hydrothermal synthesis, template, and electrochemical methods are the most commonly used to prepare TiO_2_ nanomaterials, they all require high temperatures, high pressures, and harmful chemicals to complete, limiting their synthesis and potential medical applications [83]. Green synthesis, a naturally adaptable, environmentally friendly and cost-effective technique for large-scale nanoparticles synthesis, has attracted great interest in synthesizing TiO_2_ in a green and sustainable manner [84,85]. Figure 2 shows a schematic diagram of the preparation process of nanoparticles via the green synthesis method.

Biologically active components present in organisms such as plants and bacteria promote bio-reduction and capping processes. A range of natural reducing agents, including proteins, enzymes, and phytochemicals, are involved in the synthesis of TiO_2_ [83]. Kaur, H. et al. synthesized TiO_2_ with a mesoporous nature using Titanium (IV)-iso-propoxide as a precursor and *Lagenaria siceraria* leaf extract as a reducing and capping agent. They also compared the optical, morphological, structural and photocatalytic properties of green and chemically synthesized TiO_2_. The photocatalytic decolorization of RG-19 dye was successfully enabled by green synthesized TiO_2_ using *L. siceraria aqueous* leaf extract [86]. Furthermore, the immobilization of silver nanoparticles (Ag NPs) on the TiO_2_ surface was carried out using *Carpobrotus acinaciformis* extract as a reducing and stabilizing agent. The modified TiO_2_ was characterized by FT-IR spectroscopy, X-ray diffraction, field emission scanning electron microscopy and energy dispersive X-ray spectroscopy, and it was confirmed that the diameter of Ag NPs on the TiO_2_ surface was in the range of 20-50 nm. Photocatalytic degradation experiments of methyl orange and Congo red proved that the green synthesized composite was a highly active and recyclable photocatalyst [87]. The various green sources have been used for TiO_2_ synthesis, as reported in Table 3, which gives a broader view of the green approach. The synthesis of inorganic nanoparticles using biological entities is of great interest due to their unusual optical, chemical, photo electrochemical and electronic properties [88]. *Bacillus sphaericus* was well adapted to heavy metals and could produce unusual inorganic nanoparticles by intracellular or extracellular mechanisms [89]. Suriyaraj S. P. et al. reported a one-pot method for producing TiO_2_ with pure anatase phase utilizing the extremophilic bacterium *Bacillus licheniformis*. Without calcination, the TiO_2_ particles had excellent crystalline characteristics [90]. Microbial-mediated synthesis of TiO_2_ nanoparticles was also carried out using *Aeromonas hydrophila* and *Bacillus subtilis* [91,92]. TiO_2_ produced by fungi, like bacteria, has safety limits. Nonpathogenic strains, on the other hand, will eliminate the threat and may be commercially exploited [93]. Table 4 shows the synthesis of TiO_2_ by various bacterial species.

### 2.7. Other Methods

In addition to innovations in traditional preparation methods, researchers are currently also exploring several new preparation methods. Recently, a study reported a new method based on a microwave–ultrasound-assisted method for the preparation of defective TiO_2_ and WO_3_/TiO_2_ nanoparticles. The method required only 5 min of microwave and ultrasonic irradiation to obtain TiO_2_ with a microcrystalline size of about 6 nm [109]. A study comparing the ability of ZnO-TiO_2_ nanocomposites synthesized by ultrasound-assisted and conventional methods to degrade methylene blue dye showed that ultrasound-synthesized ZnO-TiO_2_ nanocomposites exhibited superior photocatalytic activity and that the degradation process followed a secondary kinetic model [110]. Studies have also attempted the preparation of TiO_2_ nanoparticles by inter-cation redox reaction, anode synthesis by the boosting method, current hydrodynamic transport, and one-step catalyst-free gas-phase transport [111,112,113,114].

## 3. Functionalization of TiO_2_

**Figure 3:**
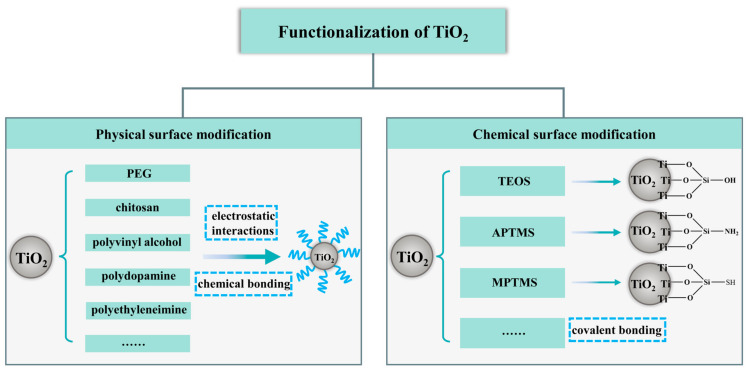
Functionalization of TiO_2_ nanoparticles.

The physiochemical characteristics of TiO_2_, like biocompatibility and functional modification, offer an excellent nanoplatform for drug delivery [45]. In particular, is it a perfect building block of novel bioinorganic hybrid nanoconjugates, which show enhanced features for imaging, photocatalytic therapy and photoactivated drug release [20,115,116]. The presence of a large number of free hydroxyl groups on the surface and inside the pores facilitates the functionalization of TiO_2_ [117]. Several techniques have been used to attach organic or inorganic ligands to the surface of TiO_2_ in order to provide advantages such as slow or controlled-release molecular delivery, improved biocompatibility, increased targeting activity, and much greater stability [14,22,118,119,120]. On the basis of surface functionalization, TiO_2_ offers the possibility to act as nanocarriers to selectively deliver drugs to specific sites, allowing drugs to exert their therapeutic effects with fewer side effects and improved therapeutic efficacy [121]. Figure 3 shows the functionalization of TiO_2_ nanoparticles.

### 3.1. Physical Surface Modification

Physical surface modification can be achieved by covering the nanoparticle surface with an ionic or polymeric surfactant. The chemical structure of surfactants contains hydrophilic and hydrophobic ends, and the hydrophilic end is adsorbed on the surface of TiO_2_ through electrostatic interactions or chemical bonding for the purpose of modification, which can reduce the inter-particle interactions and minimize the influence of interfacial forces, thus improving the stability of the particles [122].

In recent years, many researchers have reported surface modification of TiO_2_ using surfactants. Polyethylene glycol (PEG) has become a more commonly used modified surfactant because of its FDA-approved and inexpensive nature, improved hydrophilicity, improved dispersion of nanoparticles, and characteristics that allow for favorable pharmacokinetics and tissue distribution [123,124]. Furthermore, PEG can interfere with the contacts of the mononuclear phagocyte system and therefore reduce the clearance of nanocarriers [123]. A study employing atomic and molecular dynamics simulations investigated the grafting of PEG onto the surface of TiO_2_ in various solvents. They discovered that spherical brushes with high grafting density in water, but not in dichloromethane, follow the Daoud–Cotton classical scaling model for polymer volume fraction dependence with distance from the center, which has been developed to describe star-shaped polymer systems [125]. Sun et al. investigated the effect of average molecular weight of PEG on the stability and re-dispersibility of TiO_2_ dispersion. Through hydrogen bonding, PEG molecules were adsorbed on the surface of TiO_2_. As the average molecular weight of PEG molecules increased, the dispersion stability and re-dispersibility of TiO_2_ progressively improved due to their distinct adsorption conformations. However, PEG molecules with a high molecular weight can reduce nanoparticle dispersion stability [126]. In addition to PEG, chitosan [127], polyvinyl alcohol [128], polydopamine [129] and polyethyleneimine [130] are also commonly used for modification.

### 3.2. Chemical Surface Modification

Chemical surface modification is an effective method for developing surface properties of nanomaterials, and this technique is based on covalent bonding between the modifier and the particle surface [122]. Numerous studies have been conducted on the chemical modification of nanoparticle surfaces, and coupling agents such as organic functional silanes [131], polymers [132], and carboxylic acids [133,134] are frequently used in the modification process.

Silica coating is a typical chemical approach for the surface modification of TiO_2_, providing various benefits such as long-term stability, biocompatibility, and the hydrophilicity of silane-modified TiO_2_. The Stöber method is the most traditional method for silica modification of TiO_2_. This reaction typically uses anhydrous ethanol as the reaction medium, tetraethoxysilane (TEOS) as the silica source, and ammonia as an alkaline catalyst to control the hydrolysis of TEOS and the thickness of the silica layer, resulting in the formation of TiO_2_@SiO_2_ nanoparticles with regular morphology [14]. The modification techniques are continuously optimized, and the double template method, sol-gel method, and recycled template method have also become excellent methods to consider for TiO_2_@SiO_2_ [135,136,137].

The TEOS-modified TiO_2_ surface has Si-O bonds, which are derived from the silica shell. However, for other modifications with specific needs, it is necessary to introduce new groups on the surface of TiO_2_. Through the modification of different kinds of silanes, the surface of TiO_2_ is introduced to different groups, which is favorable for the next step of drug-controlled-release system. Shi et al. employed (3-aminopropyl) triethoxysilane (APTMS) to react with TiO_2_ in a toluene medium, converting the surface groups of TiO_2_ to amino groups. The TiO_2_ with amino surface was then modified to create a unique ROS-sensitive drug delivery system, which could effectively encapsulate Docetaxel (DTX) and demonstrate good acoustic kinetic chemotherapeutic effects in both in vivo and in vitro experiments [138].

(3-Mercaptopropyl) trimethoxysilane (MPTMS) is an excellent choice for introducing sulfhydryl groups to TiO_2_, and it is especially well-suited to the chemical attachment of metal nanoparticles to TiO_2_. Recently, it was reported that Ag NPs were modified on the surface of the TiO_2_, which was functionalized with MPTMS. In the presence of NaBH_4_, Ag NPs@MPTMS-TiO_2_ showed ultra-high catalytic activity for 4-nitrophenol reduction, with an apparent kinetic rate constant (k_app_) of up to 394 × 10^−3^·s^−1^ [139]. Furthermore, TiO_2_ modified with MPTMS was able to overcome the tough problem of inadvertent agglomeration of gold nanoclusters deposited on its surface, providing an effective reference for photocatalytic systems [140].

## 4. Sustained Drug Delivery

Sustained drug delivery systems (SDDSs) have been intensively studied for decades with some success in the pharmaceutical field. These can prolong the therapeutic effect by slowly releasing therapeutic substances after administration, thereby reducing the frequency of administration and minimizing side effects. The ideas of TiO_2_ carriers used for SDDS have been categorized into three groups: pore structure and morphology, interaction force with drugs, and diffusion inhibition effect.

### 4.1. Pore Structure and Morphology

The pore structure and morphology of the carrier have a significant impact on the sustained release rate of drugs. Therefore, in order to achieve drug sustained release, it is necessary to design TiO_2_ with non-connected pore structures or relatively small pore sizes. Mesoporous titanium dioxide (MTN), with pore sizes ranging from 2 to 50 nm, has controllable porosity at the mesoscale that allows for tunable release rates for drug delivery applications [141]. The drug release properties of MTN and zirconium dioxide were investigated using amoxicillin as a drug model. The drug release kinetics of both nanocarriers were governed by a diffusion control mechanism, and both could deliver the drug continuously for more than 28 days. In contrast, the slightly better drug release rate of MTN was due to its microstructure that retains the drug better [142]. Hollow nanostructures are also often studied due to their high surface area, low density and good permeability. Their inner cavities can accommodate a large number of guest molecules and the porous shells can be used as channels for drug release [143,144,145]. Liu prepared titanium dioxide hollow nanoparticles (HTNs) with controllable particle size by the hydrothermal method, and they also investigated the effects of parameters such as stirring time, precursor concentration, and hydrothermal time on the synthesis. It was found that the prepared HTNs exhibited pH-responsive slow-release behavior in vitro. The release of the loaded drug at different pH (7.4, 6.5, 5.0) conditions increased with decreasing pH value and showed a slow and constant trend, which was exemplified by the zero-order kinetic equation [143]. In addition, the plush TiO_2_ nanospheres obtained from the preparation had a high surface area, which was favorable for drug loading and easier for the drug to enter the active site. The drug (doxorubicin), before reaching dynamic equilibrium, was released continuously by diffusion control [45,146,147,148,149].

### 4.2. Interaction Force

The sustained release of the cargo could be achieved by taking advantage of the strong interaction forces such as electrostatic interactions and hydrophobic interactions between the carrier and the cargo. In the drug release behavior of mesoporous TiO_2_ films loaded with ibuprofen (IBU), the highly loaded films were able to release for a long period of 30 h. This result was not only related to the worm-like curved pores of the TiO_2_ films, but the hydrogen-bonding interactions between the IBU and the TiO_2_ films also played a crucial role [150]. The HTNs were bound to the drugs through electrostatic interactions. The release studies also revealed that the drugs loaded in HTNs were released for a longer period and could produce long-term antimicrobial efficacy [151]. In addition, the anticancer drug Daunorubicin (DNR) has three potential metal-binding sites capable of forming complexes with TiO_2_. The surface of TiO_2_ is negatively charged in an aqueous solution at pH 7.4, whereby the positively charged DNR self-assembles onto the TiO_2_ surface via electrostatic interactions. This pH-responsive novel DDS release behavior is capable of prolonging the retention time of the drug in the blood circulation, greatly reducing the side effects on normal tissues, and thus significantly improving the effectiveness of cancer therapy [152].

### 4.3. Diffusion Inhibition Effect

Modification of polymers on the surface of TiO_2_ increases the diffusion distance and provides hindrance, thus enabling a more sustained drug release. Polydopamine with multifunctional groups is a popular polymer modifier. Titanium dioxide nanotubes (TNTs) functionalized with polydopamine (PDA) were prepared using ultrasonication methods, and their excellent self-polymerization ability can effectively enhance drug loading and prolong drug release [22]. In addition, TNTs modified with (3-Glycidoxypropyl) trimethoxysilane (GPTMS) also exhibited prolonged release of dexamethasone (DEX). It was the electrostatic and hydrogen-bonding effects of the epoxy ring provided by GPTMS that stabilized the drug molecules on the surface of the TNTs, allowing for a slow and sustained release compared to unmodified TNTs [120].

## 5. Controlled Drug Delivery

**Figure 4:**
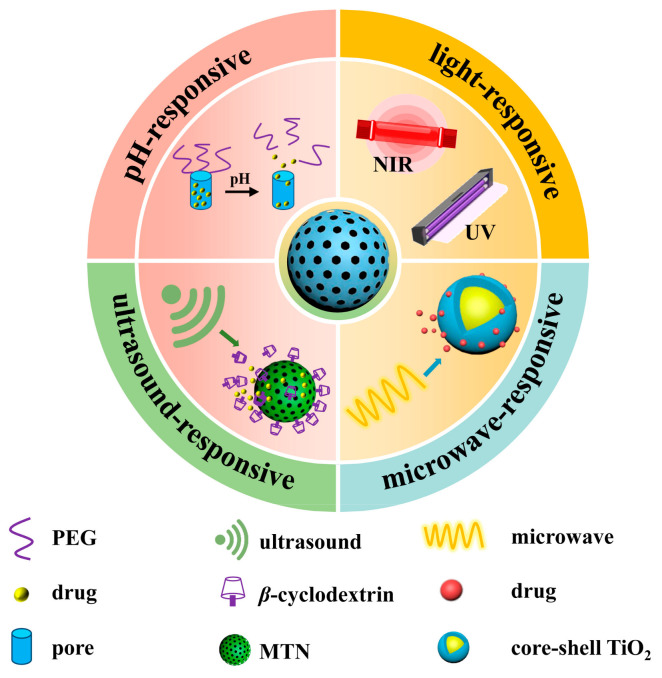
Schematic diagram of drug-controlled-release delivery systems with different responses.

**Figure 5:**
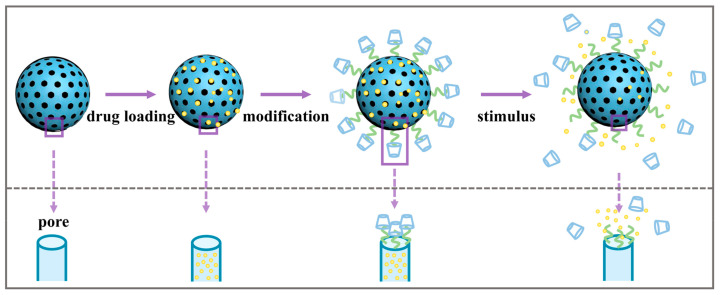
Schematic diagram of drug release process triggered by stimulation.

To prevent the premature release of drugs in the carrier, which would result in reduced efficacy and side effects, TiO_2_-based controlled-release drug delivery systems have been developed to ensure the pharmacological effects while improving drug bioavailability and enhancing the target specificity of drugs. Such controlled drug delivery systems (CDDSs) are usually achieved through modifying various “gatekeepers” via physical adsorption or covalent bonding interactions, where the drug is blocked inside the pore until the CDDSs are exposed to specific stimuli such as pH, enzymes, and redox. In addition to the above endogenous stimuli, controlled release of drugs can also be achieved by relying on exogenous stimuli (light, ultrasound, magnetic, etc.). Figure 4 and Figure 5 show schematic diagrams of drug-controlled-release delivery systems with different responses and triggering the drug release process.

### 5.1. pH-Responsive

Due to the fast growth of cancer cells, there is a hypoxic environment and lactic acid generation via glycolysis, which lowers the extracellular pH of cancerous cells. This phenomenon, known as the Warburg effect, has led to pH-responsive drug delivery platforms [153]. Furthermore, TiO_2_ nanotube-based pH-controlled-release drugs and their release kinetics are frequently researched [154,155].

However, for the construction of pH-intelligent CDDSs, it is common to utilize the surface modification of the TiO_2_ to achieve the release in a desired set of environments. Some polymers are considered effective pH-responsive modifiers. For example, the use of alendronate sodium trihydrate (AST)-modified mesoporous titanium dioxide nanoparticles (MBTNPs) as carriers for DEX drug delivery was also able to achieve pH sensitization, which relies on hydrogen-bonding interactions between the amino groups on the surface of MBTNPs and the hydroxyl groups of DEX [156]. The poly (acrylic acid)-calcium phosphate (PAA-CaP) composite layer has pH-responsive solubilization properties, and TiO_2_ modified in this way accumulates much faster doxorubicin (DOX) release at pH5.6 than at pH7.4. Furthermore, the PAA-CaP layer exhibits the higher loading and encapsulation capability of DOX [157]. In another study, TNTs were first coated with dopamine, resulting in a dopamine surface with nanoparticles containing amino groups, and then polymethylsilicic acid (PMAA) was covalently attached to the surface via carboxyl–amino group interactions. PMAA has been observed to inflate at pH = 7 and collapse at pH ≤ 6 [158]. Thus, PMAA functioned as a pH-sensitive and switchable molecular “gate” for long-term and on-demand drug release [159]. Other polymers that have been used in the construction of pH-controlled-release platforms include polyvinylpyrrolidone (PVP) [160] and polyethyleneimine (PEI) [161].

### 5.2. Light-Responsive

A theoretical basis for light-triggered drug release is provided by the fact that the energy absorbed from the ultraviolet (UV) surpasses the band gap of TiO_2_, which causes the valence electrons to be excited toward the conduction band, forming an electron (e^−^) and hole (h^+^) pair and further generating active free radicals (OH· and O^2−^) [162,163]. The novel hybrid PEI modified porous TiO_2_ nanomaterials were developed by researchers. PEI could form a hydrophilic coating on the surface of TiO_2_ and prevent premature drug release. After UV irradiation, the PEI coating was destroyed by free radicals, and the encapsulated drug molecules were thus released, realizing a UV-triggered drug delivery system [20]. In addition, it is reported that triggering chitosan (CS) can also control drug release in response to UV irradiation. CS is a natural linear polysaccharide and it has been widely used in pharmaceutical and biomedical applications due to its unique biocompatibility, biodegradability and other properties [164,165]. To improve and control drug delivery to tumors, methotrexate (MTX) loaded in CS nanoparticles has been previously developed. Al-Nemrawi et al. innovatively combined TiO_2_ with MTX-CS, and TiO_2_ was used to trigger polymer bond breaking of CS nanoparticles to enable on-demand drug release through photodegradation. Compared to pure CS, MTX, and TiO_2_, the system had a strong effect against MCF-7 breast cancer cells, with viabilities as low as 7% [166]. Recently, the same study prepared a triggerable system with a core–shell structure, using CS-modified TiO_2_ as a microcapsule loaded with oregano essential oil. It was observed that the shell molecules adsorbed TiO_2_ uniformly through hydrogen and ligand bonds. Since the free radicals generated by TiO_2_ under the action of UV light degraded the glycosidic bonds of CS, loading TiO_2_ on microcapsules could achieve UV-triggered release of external stimuli, and the release process followed the classical Fickian diffusion mechanism [127]. This system is an effective strategy for controlling the release by UV irradiation as needed, which can be applied to biomedical applications.

However, UV light is known to be harmful and there are some limitations in its application [167]. Near-infrared (NIR) light can penetrate deeper into tissues than UV light, reducing cell damage. As a result, an NIR-stimulated responsive drug delivery system based on TNTs has been proposed. For example, black phosphorus (BP) was introduced into the TNTs and 1-tetradecanol acted as a “gatekeeper” to stop drug leakage. Due to the photothermal properties of the composite, the temperature of the drug delivery system increased after irradiation with near-infrared light, reaching the melting point of the “gatekeeper” leading to its decomposition, and the blocked drug could be released [168]. To suit various therapeutic needs, NIR-stimulated responsive drug delivery systems are capable of transporting not only drugs but also cytokines to establish an ideal local immune microenvironment [169]. It has also been shown that the TiO_2_ surface was capable of attaching enzymes and light-inducing their release [170].

### 5.3. Microwave-Responsive

According to research, TiO_2_ may effectively absorb microwave energy and transform it into localized heat, creating a microwave-induced thermal effect that is sensitive and selective [171,172]. In a study, multifunctional Fe_3_O_4_@TiO_2_: Er^3+^, Yb^3+^-glycine nanocomposites were prepared using a hydrothermal method. Thermodynamic analyses demonstrated that the interaction between the carrier and the drug etoposide occurred through relatively weak hydrogen bonding, which was easily broken by microwave irradiation [173]. Cui’s group constructed Janus-shaped TiO_2_-x&mSiO_2_ nanoparticles using gray-black titanium dioxide (TiO_2_-x) and mesoporous silica (mSiO_2_) as carriers. The rod-shaped mSiO_2_ acted as an efficient drug carrier, while TiO_2_-x served as the main microwave absorber. DOX and Janus TiO_2_-x&mSiO_2_ interacted with each other by van der Waals forces. Under the stimulation of microwave, the carrier enhanced the drug release rate with excellent thermal conversion ability. Meanwhile, the drug release experiments showed a significant pH-dependent release behavior. Cytotoxicity experiments verified the good biocompatibility of Janus TiO_2_-x&mSiO_2_, and this drug delivery carrier was expected to pave the way for subsequent applications [174]. Recently, the authors constructed “Biped” Janus Fe_3_O_4_@nSiO_2_@TiO_2_-x&mSiO_2_ nanoparticles as drug carriers, which also possessed pH and microwave dual-triggering properties [175].

### 5.4. Ultrasound-Responsive

Ultrasound is a promising method for controlling medication release from some carriers. Ultrasound waves can be precisely targeted onto a specific area, and the interaction of ultrasound with tissues can produce a variety of beneficial bioeffects [176]. Cavitation produced by ultrasound is a well-known ultrasonic effect, leading to the development, growth, and burst of gas/vapor-filled microbubbles in liquids. During cavitation, the collapse of the bubble is adiabatic, and thus the bubble serves to concentrate acoustic energy to locally generate extreme temperature and pressure conditions in a short period [177]. Currently, there are various ultrasound responsive drug delivery vehicles based on TiO_2_ nanomaterials [178,179,180]. A multifunctional capsule system with a core–shell structure capable of simultaneous fluorescence imaging, magnetically guided delivery, and ultrasound-triggered release was reported [181]. The system used safe olive oil as a reservoir for oil-soluble drugs, and Fe_3_O_4_, carbon quantum dots, and bilayer porous TiO_2_ shells as sensitive carriers. The capsule cracked more and more as the ultrasonic duration increased, until the porous titanium shell broke and the medication gushed out. It was demonstrated that the ultrasound period might regulate the drug’s release profile.

An ideal DDS should be able to effectively encapsulate the drug before it reaches the target site [182]. As a result, it is necessary to cover the carrier surface with an effective “gatekeeper”. The β-Cyclodextrin (β-CD) was a typical “gatekeeper”, which can effectively capture a variety of payloads [183,184,185]. One study applied β-CD to drug-loaded MTN as a massive “gatekeeper” to block drug leakage from the mesopore. To enable a burst of drug release, β-CD was attached to the surface of MTN via a ROS-sensitive linker (HOOC-S-CH_2_-S-COOH). Once the ROS-sensitive linker was recognized and destroyed by ROS, the drug (DTX) in the carrier (MTN@DTX-CD) was instantaneously released. In vivo and in vivo studies demonstrated that this DDS showed good ultrasound-triggered effects and the feasibility of sonodynamic chemotherapy [138]. Previous studies have revealed that PEI could not only serve as “gatekeeper” by blocking mesopore pores, but also increase the cellular uptake of silica mesoporous nanoparticles (MSNs) and develop a safe and effective drug delivery system. Based on this, dendritic silica/titanium dioxide mesoporous nanoparticles (DSTNs) with PEI as a “gatekeeper” and folic acid (FA) as a targeting agent were reported. Ultrasound led to the generation of ROS from TiO_2_, which destroyed the “gatekeeper”, resulting in the mesoporous pores being unblocked and the curcumin (Cur) being released. The results demonstrated that the Cur@PEI-FA-DSTNs system performed better in combined chemo-acoustic-dynamic cancer therapy than in acoustic-dynamic therapy or chemotherapy alone [186].

### 5.5. Dual- or Multiple-Stimuli-Responsive

Nowadays, the therapeutic performance of single-responsive carriers is hardly satisfactory, and the integration of multiple stimulus-response mechanisms into one carrier is an extremely promising strategy [187]. According to previous research, Janus-shaped TiO_2_-x&mSiO_2_ nanoparticles and “Biped” Janus-Fe_3_O_4_@nSiO_2_@TiO_2_-x&mSiO_2_ nanoparticles could not only release drugs under pH control, but also be further triggered by microwave stimulation [175,178]. A novel composite material in which TiO_2_ and SiO_2_ nanostructures were synergistically assembled on the surface of the polymer shell of a microcapsule was capable of achieving a triple response to enzyme, UV and ultrasound. It was reported that UV and ultrasound irradiation caused the breakage of SiO_2_/TiO_2_-coated capsules into small fragments and the enzymatic degradation led to the deformation of the capsules and cargo leakage [188]. We summarized the DDSs of TiO_2_ in Table 5.

## 6. Conclusions and Outlook

In this review, we summarize research advances in TiO_2_ nanomaterials in terms of preparation methods, surface functionalization and drug delivery. Compared with organic nanomaterials such as liposomes, TiO_2_ nanomaterials have the advantages of easy functionalization, controllable particle size and morphology, stable physicochemical properties, and high loading capacity. The unique photocatalytic nature of TiO_2_ nanomaterials endows them with potential functions such as photothermal therapy, which can be considered a rising star in the field of biomedicine and an ideal therapeutic nanocarrier [189]. The high versatility of these smart nano-delivery systems opens up great prospects for the innovation of novel nanomedicines and provides an effective strategy for personalized therapy and diagnostics.

Although TiO_2_-based smart systems have made great progress, there is still much potential for development. On the one hand, the most studied stimuli for TiO_2_-based nanoparticles in the design of controlled-release drug delivery systems are pH, light, ultrasound, and microwave, while single-response delivery systems such as redox response and enzyme response, and dual-response delivery systems are rarely reported. These endogenous responses can be the future development direction for TiO_2_-based drug delivery systems. In addition, the combination of exogenous and endogenous stimuli can further enhance the spatio-temporal controllability as well as the accuracy of nanomaterials’ delivery, and multi-stimulus-responsive nanomaterials have significant advantages in vivo cycling, tumor retention, tissue penetration, cellular internalization, and endosomal escape, which will be the focus of future research.

On the other hand, it is obvious that a large amount of work still needs to be carried out before drug delivery systems achieve clinical translation. However, some TiO_2_ nanoparticles have been found to have several adverse effects on highly sensitive stem cells, with inflammatory responses being an important feature of their cytotoxicity [190,191]. The data emphasized the correlation of factors such as particle size, shape and crystal structure with the level of genotoxicity [192,193]. Mechanistically, the observed adverse reactions were associated with upregulation of p38, JNK and ERK protein expression [190]. The European Commission has already banned the use of TiO_2_ (E171) as a food additive from August 2022, citing genotoxicity. Therefore, we must pay more attention to the potential cytotoxicity of the TiO_2_ nanoparticles at the time of application. Long-term toxicity tests and tolerance studies need to be performed on animals to evaluate the safety of blank DDS and drug-loaded DDS in depth before proceeding with human clinical trials. More in-depth studies should be carried out by optimizing various process parameters to select TiO_2_ nanoparticles that are more reliable and more suitable for biomedical applications. In addition, the examination of DDS immunoreactivity is also crucial to evaluate the safety of nanoparticles. Repeated exposure to materials that are often considered biocompatible and non-immunogenic can also lead to the production of antibodies that may trigger hypersensitivity reactions [194]. Given that long-term exposure to nanoparticles may cause significant immunogenic effects, comprehensive in vivo studies are essential. Regarding the potential issues with TiO_2_ nanoparticles, researchers have been actively addressing them with little success. We believe that some TiO_2_ nanoparticles still have broad prospects in the biomedical field.

## Figures and Tables

**Table 1:** Advantages and disadvantages of different types of inorganic nanomaterials.

Type	Advantages	Disadvantages	Application
TiO_2_	Easy surface functionalization, antibacterial properties, photocatalytic degradation properties	Wide band gap, fast hydrolysis rate and lack of biosafety evaluation	Nano-biosensing, medical implantation, drug delivery, and antimicrobial agents
SiO_2_	Low toxicity, easy surface functionalization	Lack of biosafety evaluation	Bioimaging, drug delivery
Fe_3_O_4_	Magnetic, biodegradability	Easy to aggregate and oxidize	Biological separation and detection, magnetic resonance imaging, and magnetic hyperthermia

**Table 2:** Characteristics of different synthesis methods.

Methods	Classification	Characteristic
Sol-gel method	-	Simple, fast, economically less expensive, low processing temperature, homogeneity of the produced material
Hydrothermal method	-	High purity, good dispersibility, low safety and high energy consumption
Template method	Hard template method	Simple operation and various structures
	Soft template method	
Gas-state method	Physical vapor deposition	High purity, uniform distribution, small particle size and good dispersion, expensive
	Chemical vapor deposition	High purity, high reaction temperature
Solid-state method	-	Easy to operate, short processing time, impurity, incomplete morphology and inhomogeneous particle size
Green synthesis method	-	Naturally adaptable, environmentally friendly and cost-effective

**Table 3:** TiO_2_ prepared by utilizing a variety of plants.

S/N	Plant Extract	Reactant	Shape	Average Particle Size (nm)	Application	Ref.
1	*Tinospora cordifolia*	Titanium (IV) isopropoxide	Triangular and irregularly shape	7–21	Photocatalysis	[94]
2	*Citrus Limetta*	Titanium butoxide, water	Spherical	80–100	Photocatalysis	[95]
3	*Syzygium cumini*	Titanium-isopropoxide, water	Spherical	15–22	Photocatalysis	[96]
4	*Luffa acutangula*	Titanium sulfate, water	Aggregated	10–49	Antibacterial	[97]
5	*Mentha arvensis*	Titanium tetra-isopropoxide, ethanol	Spherical	20–70	Antibacterial	[98]
6	*Acorus calamus*	Titanium (IV) isopropoxide, water, aqueous ammonia	Spherical and interconnected	11–30	Photocatalysis and antibacterial	[99]
7	*Citrus limon*	Titanium (IV) butoxide, isopropanol, glacial acetic acid	Spherical	9–18	Dye-sensitized solar cells	[100]
8	*Curcuma Longa L.*	Methanol, titanium tetra-isopropoxide, water	Spherical, cubic and hexagonal	20.8–40.1	Photocatalysis	[101]
9	*Phyllanthus niruri*	Titanium isopropoxide, water	Spherical	20	Dye adsorption	[102]
10	*Laurus nobilis*	Titanium tetra isopropoxide, water	Spherical	80–120	Antibacterial and antioxidant	[103]

**Table 4:** TiO_2_ produced by several bacterial communities.

S/N	Bacterial Species	Reactant	Shape	Average Particle Size (nm)	Application	Ref.
1	*Aeromonas hydrophila*	Metatitanic acid (TiO (OH)_2_)	Spherical	40–50	Antibacterial	[104]
2	*Lactobacilli*	TiO (OH)_2_	Irregular	10–25	-	[105]
3	*Bacillus subtilis*	TiO (OH)_2_, water	Spherical	66–77	-	[106]
4	*Bacillus subtilis*	Ti^4+^ ions, water	Spherical	10–30	Photocatalysis	[92]
5	*Alcaligenes aquatilis*	K_2_TiF_6_, water, silver nitrate solution	Spherical	10–90	Photocatalysis	[107]
6	*Fusarium oxysporum*	Aqueous solution, (CH_3_COO)_2_Ba, K_2_TiF_6_	Spherical	10	-	[108]

**Table 5:** TiO_2_ drug delivery systems with different response types.

TiO_2_-Type	Synthesis Method	Drug	Stimulus	Ref.
MTN	Polymer sacrificial method	Amoxicillin	Sustained release	[142]
HMTN	Hydrothermal method	Doxorubicin	Sustained release	[143]
HMTN	Stöber method	Curcumin	UV	[144]
HMTN	Surface-layer-absorption template	1,2-benzisothiazolin-3-one	Sustained release	[145]
plush TiO_2_	Hydrothermal method	Doxorubicin	Sustained release	[45]
TiO_2_ film	EISA method	Ibuprofen and vancomycin	Sustained release	[150]
HMTN	Sol-gel method	Gentamycin	Sustained release	[151]
DNR-TiO_2_	-	Daunorubicin	pH	[152]
PDA-TNTs	-	Ibuprofen	Sustained release	[22]
GPTMS-TNTs	Hydrothermal method	Dexamethasone	Sustained release	[120]
TNTs	-	Doxorubicin	pH	[154]
TNTs	Two-step anodization method	Methotrexate	pH	[155]
MBTNPs	Template method	Dexamethasone	pH	[156]
TiO_2_@PAA-CaP NPs	Hydrothermal method	Doxorubicin	pH	[157]
PVP-Ag-TiO_2_	-	Doxorubicin	pH	[160]
TiO_2_@Fe_3_O_4_-PEI	Sol-gel method	Doxorubicin	pH	[161]
CS-NPs	Ionic gelation method	Methotrexate	Light	[166]
BP-TNTs	-	Ibuprofen	NIR	[168]
BP-TNTs	Electrochemical reduction method	IL-4	NIR	[169]
TiO_2_	-	Proteins	UV	[170]
Fe_3_O_4_@TiO_2_	Hydrothermal method	Etoposide	Microwave	[173]
TiO_2_-x&mSiO_2_	Sol-gel method	Doxorubicin	Microwave and pH	[174]
Fe_3_O_4_@nSiO_2_@TiO_2_-x&mSiO_2_	-	Doxorubicin	Microwave	[175]
Superhydrophobic -TNTs	Tetracycline hydrochloride	Electrochemical anoDization method	Ultrasound	[178]
TiO_2_ nanosticks	-	GlioblasToma multiforme	Ultrasound	[179]
TiO_2_-x @NaGdF_4_	-	IR780 iodine	Ultrasound	[180]
TiO_2_ Core-Shell Capsules	Coaxial electrospray method	Paclitaxel	Ultrasound	[181]
PEI-FA-DSTNs	-	Curcumin	Ultrasound	[186]
MTN-CD	Sol-gel method	Docetaxel	Ultrasound	[138]
SiO_2_/TiO_2_	Template method	Rh-B	UV, ultrasound and enzymatic treatment	[188]
